# Supplementary material for: Bilirubin Exerts Protective Effects on Alveolar Type II Pneumocytes in an In Vitro Model of Oxidative Stress
Source: Int J Mol Sci. 2024 May 13;25(10):5323. doi: 10.3390/ijms25105323 (PMC11121655; doi:10.3390/ijms25105323)
Supplement: Supplementary file 1 [file ijms-25-05323-s001.zip › Table S-1 Quantitation of cell death.pdf]

Table S-1 Quantification of cell death (ICC of AEC II cells)

**hypoxia (5% oxygen)**		
**bilirubin**	**–**	**400 nM**
	269.8±12.5	102.1±20.8
**Normoxia (21% O_2)**		
**bilirubin**	**–**	**400 nM**
	100.0	68.6±7.9
**Hyperoxia (80% O_2)**		
**bilirubin**	**–**	**400 nM**
	256.3±28.7	116.6±27.1

Data are normalized to the level of AEC II cells exposed to normoxia (100%) and are presented as mean (%) ± standard error of the mean (SEM). n = 6 individual experiments/group.
