# Supplementary material for: Bilirubin Exerts Protective Effects on Alveolar Type II Pneumocytes in an In Vitro Model of Oxidative Stress
Source: Int J Mol Sci. 2024 May 13;25(10):5323. doi: 10.3390/ijms25105323 (PMC11121655; doi:10.3390/ijms25105323)
Supplement: Supplementary file 1 [file ijms-25-05323-s001.zip › Table S-2 Quantitation of apoptosis-related mediators.pdf]

Table S-2 Quantification of apoptosis-related mediators (qPCR of AEC II cells)

**hypoxia (5% oxygen)**	4 hours		24 hours	
**bilirubin**	**–**	**400 nM**	**–**	**400 nM**
Casp3	70.2±9.3	69.0±11.4	138.2±6.0	93.8±8.5
AIF	87.8±4.6	75.2±3.3	175.7±10.7	92.7±7.9
CycD2	93.8±6.0	90.8±9.0	109.9±7.4	98.1±10.6
**normoxia (21% O_2)**	4 hours		24 hours	
**bilirubin**	**–**	**400 nM**	**–**	**400 nM**
Casp3	100.0±2.1	82.2±7.7	100.0±6.6	102.7±12.5
AIF	100.0±4.7	91.9±7.0	100.0±8.0	96.2±8.8
CycD2	100.0±8.1	92.8±8.0	100.0±6.5	99.2±7.9
**hyperoxia (80% O_2)**	4 hours		24 hours	
**bilirubin**	**–**	**400 nM**	**–**	**400 nM**
Casp3	62.9±6.4	65.8±9.2	140.3±7.1	84.9±10.4
AIF	89.4±8.1	68.1±7.3	156.1±6.8	87.7±6.6
CycD2	91.1±8.0	93.9±6.7	63.4±4.0	183.3±12.9

Data are normalized to the level of AEC II cells exposed to normoxia (100%) and are presented as mean (%) ± standard error of the mean (SEM). n = 5 individual experiments/group.
