# Supplementary material for: Bilirubin Exerts Protective Effects on Alveolar Type II Pneumocytes in an In Vitro Model of Oxidative Stress
Source: Int J Mol Sci. 2024 May 13;25(10):5323. doi: 10.3390/ijms25105323 (PMC11121655; doi:10.3390/ijms25105323)
Supplement: Supplementary file 1 [file ijms-25-05323-s001.zip › Table S-3 Quantitation of oxidative stress-related mediators.pdf]

Table S-3 Quantification of oxidative stress-related mediators (qPCR of AEC II cells)

**hypoxia (5% oxygen)**	4 hours		24 hours	
**bilirubin**	**–**	**400 nM**	**–**	**400 nM**
Nrf2	109.3±7.0	85.4±6.6	152.6±15.6	105.1±8.8
Keap1	69.5±2.9	76.0±5.6	89.5±8.7	261.3±15.8
GCLC	93.0±3.0	78.6±3.9	150.3±8.8	102.9±30.1
Hif1α	134.8±5.3	85.9±4.9	168.3±10.5	117.9±2.1
**normoxia (21% O_2)**	4 hours		24 hours	
**bilirubin**	**–**	**400 nM**	**–**	**400 nM**
Nrf2	100.0±6.6	83.1±5.5	100.0±9.2	97.1±11.9
Keap1	100.0±7.6	80.8±4.4	100.0±12.0	103.5±8.0
GCLC	100.0±6.6	78.1±10.0	100.0±13.4	90.9±14.7
Hif1α	100.0±6.3	93.9±5.3	100.0±12.2	94.7±11.8
**hyperoxia (80% O_2)**	4 hours		24 hours	
**bilirubin**	**–**	**400 nM**	**–**	**400 nM**
Nrf2	90.9±3.9	81.8±2.1	181.8±11.11	118.0±9.0
Keap1	64.5±6.0	71.6±3.1	69.3±4.2	128.3±3.7
GCLC	124.5±2.0	79.5±4.6	138.7±5.6	85.8±4.9
Hif1α	95.0±5.3	92.4±5.0	107.7±4.5	104.3±10.1

Data are normalized to the level of AEC II cells exposed to normoxia (100%) and are presented as mean (%) ± standard error of the mean (SEM). n = 5 individual experiments/group.
