# Supplementary material for: Bilirubin Exerts Protective Effects on Alveolar Type II Pneumocytes in an In Vitro Model of Oxidative Stress
Source: Int J Mol Sci. 2024 May 13;25(10):5323. doi: 10.3390/ijms25105323 (PMC11121655; doi:10.3390/ijms25105323)
Supplement: Supplementary file 1 [file ijms-25-05323-s001.zip › Table S-4 Quantitation of inflammation-related mediators.pdf]

Table S-3 Quantification of inflammation-related mediators (qPCR of AEC II cells)

**hypoxia (5% oxygen)**	4 hours		24 hours	
**bilirubin**	**–**	**400 nM**	**–**	**400 nM**
NFκB1	100.9±8.7	82.5±4.8	191.7±6.4	122.4±14.9
NFκB2	102.7±9.9	91.4±9.8	167.0±10.9	155.4±9.1
TNFα	n.d.	n.d.	190.8±20.4	88.4±15.6
TGFβ	94.4±11.6	74.1±6.5	194.5±14.9	123.8±16.6
**normoxia (21% O_2)**	4 hours		24 hours	
**bilirubin**	**–**	**400 nM**	**–**	**400 nM**
NFκB1	100.0±5.1	94.2±9.7	100.0±11.7	87.7±7.6
NFκB2	100.0±4.7	89.4±4.3	100.0±9.2	92.6±1.8
TNFα	n.d.	n.d.	100.0±11.5	108.5±13.0
TGFβ	100.0±8.5	77.6±6.7	100.0±9.5	82.9±11.8
**hyperoxia (80% O_2)**	4 hours		24 hours	
**bilirubin**	**–**	**400 nM**	**–**	**400 nM**
NFκB1	91.2±7.6	79.4±7.3	108.6±7.4	89.7±7.9
NFκB2	91.2±5.9	72.8±4.0	134.7±2.9	102.6±7.5
TNFα	n.d.	n.d.	144.7±4.9	84.6±14.1
TGFβ	89.3±5.7	57.2±6.3	104.2±8.9	94.1±16.3

Data are normalized to the level of AEC II cells exposed to normoxia (100%) and are presented as mean (%) ± standard error of the mean (SEM). n = 5 individual experiments/group (n.d. means “not detectable”).
